# The first reported case of vincristine‐induced unilateral vocal cord palsy in an adult patient with HIV‐associated Burkitt‐like lymphoma being treated with dose‐escalated R‐EPOCH

**DOI:** 10.1002/ccr3.1964

**Published:** 2018-12-18

**Authors:** Leena T. Rahmat, Philip H. Brandt, Syed S. Ali

**Affiliations:** ^1^ Department of Oncology, Sidney Kimmel Comprehensive Cancer Center, Sibley Memorial Hospital Johns Hopkins University Washington District of Columbia; ^2^ Department of Hematology and Oncology, D’Amour Center for Cancer Care, Baystate Medical Center University of Massachusetts Springfield Massachusetts

**Keywords:** lymphoma, R‐EPOCH, vincristine, vocal cord palsy

## Abstract

Vincristine‐induced vocal cord palsy (VCP) is a rare but critical complication. Prompt recognition of VCP is imperative. Vincristine‐induced VCP is reversible, and a complete remission of a lymphoma is still feasible upon withdrawal of vincristine from the chemotherapeutic regimen early in the course of treatment.

## 1 INTRODUCTION

Vincristine‐induced vocal cord palsy (VCP) is a rare but grave complication. We report a case of a young male who was diagnosed with human immunodeficiency (HIV)‐associated stage III Burkitt‐like lymphoma, who developed acute voice hoarseness and grade 3 peripheral neuropathy following the second dose of chemotherapy with dose‐escalated rituximab, etoposide, doxorubicin, vincristine, and cyclophosphamide. Fiberoptic laryngoscopy revealed a sluggish right vocal cord confirming unilateral vocal cord paralysis. Vincristine was discontinued from subsequent cycles of chemotherapy resulting in complete resolution of the vocal cord palsy and resolution in the peripheral neuropathy over eight weeks. He had complete response (CR) following completion of chemotherapy. This case highlights that instantaneous recognition of vincristine‐induced vocal cord palsy is paramount and requires an urgent otolaryngology evaluation. Furthermore, it can be reversed and achievement of a CR is still possible with withdrawal of vincristine from the chemotherapeutic regimen early in the course of treatment.

Vincristine, a vinca alkaloid, is derived from the periwinkle plant (Vinca Rosacea) and has been used to treat numerous childhood and adult malignancies for several decades.^1^ Vincristine has a well‐established role in the treatment of hematologic malignancies and solid malignancies. Neurotoxicity is a well‐recognized adverse effect of vincristine, and vincristine results in a mixed sensorimotor neuropathy or autonomic neuropathy (bladder atony, orthostatic hypotension, paralytic ileus).^1^ It is a dose‐dependent effect that typically occurs early in the course of treatment^1^ and while the pathogenesis of vincristine‐induced neuropathy remains unclear it may be related to an effect on microtubules resulting in malorientation of microtubules and neurofilaments.^1^ Recurrent laryngeal nerve palsy resulting in vocal cord palsy (VCP) caused by vincristine has also been reported in literature, predominantly in the pediatric population.^1^, ^2^, ^3^, ^4^ Instant recognition and management of vincristine‐induced VCP is crucial because it is a potential fatal complication albeit one that can be completely reversed.

We present the first reported case of an adult male with human immunodeficiency virus (HIV)‐associated high‐grade B‐cell lymphoma who developed acute vincristine‐induced vocal cord paralysis (VCP) and peripheral neuropathy during the early course of treatment with a vincristine‐containing chemotherapeutic regimen.

## 2 CASE PRESENTATION

A 32 year‐old Caucasian male with recently diagnosed HIV was admitted with acute symptomatic microcytic anemia, fatigue, and abdominal pain. He was diagnosed with HIV four months prior to presentation and had been initiated on anti‐retroviral therapy (ART) as part of a trial. Absolute CD 4 count at diagnosis of HIV was 309 cells/mm^3^. An upper endoscopy for workup of the anemia revealed oozing ulcers in the stomach body and granular masses in the second part of the duodenum which were biopsied. Histology demonstrated a high‐grade B‐cell lymphoma, not otherwise specified (NOS) consistent with Burkitt‐like lymphoma in both sites, involving gastric and duodenal mucosa. Staging PET/CT demonstrated widespread metastatic disease, with gastric, duodenal, and small bowel wall thickening with intense FDG uptake, multiple peritoneal implants, hepatic lesions, moderate ascites, and bilateral thyroid intense FDG uptake, consistent with stage III disease. Bone marrow biopsy was negative for lymphoma.

Systemic chemotherapy with dose‐escalated R‐EPOCH (rituximab, prednisone, etoposide, doxorubicin, vincristine, and cyclophosphamide) was initiated. Twenty‐four hours after completing the second cycle of chemotherapy, he developed acute profound voice hoarseness and bilateral grade 3 peripheral neuropathy in his fingers and toes. The cumulative dose of vincristine he had received was 3.2 mg/m^2^. There was no obstruction or anatomical abnormality noted on CT neck. An urgent otolaryngology referral was made, and a fiberoptic laryngoscopy examination showed a sluggish right vocal cord fold and an incomplete glottic closure with a gap confirming a diagnosis of unilateral VCP due to vincristine. The subsequent four cycles of chemotherapy were continued with omission of vincristine, and there were no further complications. The subjective voice hoarseness completely resolved, and the grade 3 peripheral neuropathy improved to grade 1 within 8 weeks of discontinuing vincristine. The abdominal pain resolved, fatigue improved, and hematocrit showed continued improvement. A re‐staging PET/CT following completion of six cycles of chemotherapy demonstrated a complete response (CR). He remains in CR over one year following completion of chemotherapy.

## 3 DISCUSSION

Vincristine is a critical component of chemotherapeutic regimens to treat various adult malignancies including HIV‐associated B‐cell lymphomas. Similar to other chemotherapeutic agents, it comes with its own potential toxicities including neurotoxicity. Various neurotoxic presentations may present as sensorimotor neuropathy, cranial neuropathy, autonomic neuropathy, and rarely as encephalopathy. Factors that make patients susceptible to more severe neurotoxicity include hepatic impairment, hypersensitivity to vincristine, hereditary neuropathy, concomitant use of drugs such as erythromycin, allopurinol, isoniazid, phenytoin, and interactions between ART and chemotherapy.^6^ The mechanism of vincristine‐induced neuropathy remains elusive which has thwarted the development of preventive measures against this toxicity.

The majority of cases of vincristine‐induced VCP have been reported in the pediatric population.^1^, ^2^ Very few cases have been reported in the adult population.^1^ It is also noted that vincristine‐induced neurotoxicity tends to be related to dose intensity and a cumulative dose effect.^1^

Despite the unsettled mechanism of vincristine‐induced neuropathy, neuroprotective and neuroregenerative treatment efforts have been attempted. One study using animal models suggested a neuroprotective effect with erythropoietin (EPO).^3^ EPO, a well‐recognized hematopoietic factor, was noted to have numerous functions outside of the bone marrow and that recombinant human EPO (r‐Hu‐EPO) can cross the blood‐brain barrier.^3^ Interestingly, there is also evidence that glutamine may improve sensory function in vincristine‐induced neuropathy when studied in the pediatric populations.^4^

Vincristine‐induced VCP is fortunately reversible. One case study reported an older male with mantle cell lymphoma, who developed hoarseness due to bilateral VCP after the second cycle of chemotherapy with R‐CHOP. Vincristine was omitted from subsequent cycles of chemotherapy and he had full recovery of the VCP and had attained complete remission.^2^

Given the fact that our patient had underlying HIV, an HIV‐associated sensory neuropathy and vocal cord palsy was initially postulated. There is data to suggest an increased risk of autonomic neurotoxicity when vincristine‐containing chemotherapy is administered with proteasome inhibitor‐based ART. Vincristine is metabolized by the CYP 3A4 enzyme, and the use of CYP 3A4 inhibitors may result in increased levels leading to increased toxicity of peripheral and autonomic neuropathy. There is data evaluating the influence of ART on the pharmacokinetics of doxorubicin and cyclophosphamide^6^; however, no pharmacokinetic studies evaluating the interactions between vincristine and ART were identified.

## 4 CONCLUSION

Clinicians must have a high index of suspicion of vincristine‐induced VCP in a patient who complains of acute voice hoarseness. An urgent otolaryngology consultation and prompt withdrawal of the offending agent are required. VCP can be a life‐threatening event but fortunately it is a reversible complication. Our patient remains in CR one year following completion of six cycles of chemotherapy, despite omission of vincristine with cycles 3‐6. To our knowledge, this is the first reported case of vincristine‐induced unilateral vocal cord paralysis in a patient being treated for HIV‐associated Burkitt‐like lymphoma with dose‐escalated R‐EPOCH, who had full recovery of the vocal cord paralysis within eight weeks after cessation of vincristine.

## CONFLICT OF INTEREST

The authors declare that there is no conflict of interest.

## AUTHOR CONTRIBUTION

LTR: provided contributions to the design of the case report, acquired and interpreted the data, and drafted the manuscript. PHB: drafted the manuscript and revised it critically for significant intellectual content. SSA: provided conception of the case report and provided final approval of the version to be published.
